# Designing the MyoFusion Media: A Serum‐Free Medium Optimized for Bovine Satellite Cell Differentiation

**DOI:** 10.1002/bit.70237

**Published:** 2026-05-13

**Authors:** Aysenaz Tavsanli, Viktor Milkevych, Jette Feveile Young, Martin Krøyer Rasmussen

**Affiliations:** ^1^ Department of Food Science Aarhus University Aarhus DK Denmark; ^2^ Center for Quantitative Genetics and Genomics Aarhus University Aarhus DK Denmark

**Keywords:** cell culture media, design of experiments, growth factors, In vitro meat, myogenic

## Abstract

Cultivated meat production requires efficient differentiation of muscle progenitor cells into myotubes without relying on animal‐derived serum, which poses ethical and scalability challenges. This study aimed to develop a chemically defined, serum‐free medium optimized for bovine satellite cell differentiation. Using a data‐driven design of experiments approach, we systematically screened 13 growth factors and supplements previously associated with myogenesis. Fusion index served as the primary metric for differentiation efficiency, complemented by additional morphological traits. Initial screening identified platelet‐derived growth factor BB (PDGF‐BB), cytosine arabinoside, and linoleic acid as key contributors, with transforming growth factor beta (TGF‐β) included for its biological relevance. Subsequent optimization employed full factorial and central composite designs combined with response surface modeling to refine concentration ranges and evaluate interactions. The resulting formulation, termed MyoFusion, consists of DMEM supplemented with 4.5 × 10⁻⁶ mg/mL TGF‐β1, 4.66 × 10⁻⁶ mg/mL PDGF‐BB, 0.73 × 10⁻⁵ mg/mL cytosine arabinoside, and 2.07 × 10⁻⁴ mg/mL linoleic acid. Experimental validation demonstrated that MyoFusion achieved a fusion index of 65.08 ± 4.97, representing an improvement of up to 52.7% compared to serum‐based controls (2% FBS). Correlation analyses confirmed strong associations between fusion index and other differentiation traits under serum‐free conditions, indicating robust myotube formation. These findings establish MyoFusion as a promising serum‐free alternative for cultivated meat applications, supporting ethical and scalable production while maintaining differentiation efficiency comparable to or exceeding traditional serum‐containing media.

## Graphical abstract

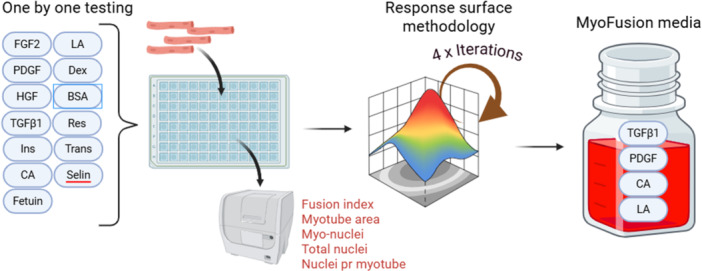

## 1 Introduction

Cultivated meat production relies on the stemness and proliferative capacity of muscle progenitor cells, particularly satellite cells (Rasmussen 2024). These cells must first undergo a proliferative phase to expand biomass before entering a differentiation phase, during which myoblasts fuse to form myofibrillar structures comparable to those found in conventional meat.

Traditionally, proliferation is supported by supplementing the culture medium with high concentrations (> 10%) of fetal bovine serum (FBS) and/or horse serum (HS). However, the use of animal‐derived serum is not acceptable in cultivated meat production due to ethical, regulatory, and scalability concerns. This underscores the urgent need to develop serum‐free media capable of supporting both the proliferation and differentiation of satellite cells.

To sustain proliferation, several signaling pathways have been identified as critical, including the fibroblast growth factor 2 (FGF2) pathway (Clegg et al. 1987). This pathway involves a canonical cascade of protein phosphorylation events initiated by activation of the FGF2 receptor. As a result, chemically defined media formulations for cultivated meat production have been developed, most of which include FGF2 and carrier proteins such as albumin (Skrivergaard et al. 2023; Stout et al. 2022; Kolkmann et al. 2020).

While chemically defined media formulations that support satellite cell proliferation are becoming increasingly available, a significant gap remains in the development of defined media specifically tailored to initiate and sustain differentiation. Nevertheless, several growth factors and supplements, previously shown to promote differentiation in other cellular systems, may hold promise for satellite cell cultures.

Induction of differentiation in the production of cultivated meat is suggested to be associated with important quality traits known from conventional meat production (Rasmussen et al. 2025; Mehmood et al. 2025). Traditionally, the induction of differentiation in cultures of satellite cells is done by exposing the cells to reduced or full serum starvation. This often leads to the deactivation of mitogenic pathways, like the MAPK pathway (Xie et al. 2018). However, the timing of maximum differentiation often varies among different batches of cells (Skrivergaard et al. 2025), which makes the future production of differentiated muscle cell biomass challenging.

FGF2 is known to regulate the growth and function of vascular cells, including endothelial and smooth muscle cells (Nugent and Iozzo 2000). Other growth factors such as platelet‐derived growth factor (PDGF‐BB), transferrin, and hepatocyte growth factor (HGF) are recognized for their mitogenic effects on muscle precursor cells. Notably, HGF and transforming growth factor beta (TGF‐β) also contribute to the chemotactic behavior of satellite cells in vitro (Morgan and Partridge 2003). Moreover, TGF‐b has been demonstrated to reduce satellite cell proliferation (Yablonka‐Reuveni and Rivera 1997), which might be fostering differentiation, possibly in combination with other growth factors. Insulin‐like growth factor (IGF) has been shown to promote differentiation, while HGF supports both migration and proliferation of tongue myogenic cells (Yamane et al. 2003). The combination of cytosine arabinoside and insulin has demonstrated improvements in the fusion index (Turo and Florini 1982), and cytosine arabinoside alone has been shown to inhibit unwanted cell proliferation (Umemoto et al. 2011). Several additional components have been identified as beneficial in serum‐free media formulations. Fetuin enhances cell attachment and growth (Skrivergaard et al. 2023; McFarland et al. 1991), while linoleic acid has been used to improve satellite cell differentiation and fusion (Allen et al. 1985). Dexamethasone has been found to enhance myotube formation (Belanto et al. 2010), and bovine serum albumin (BSA) is commonly included in serum‐free differentiation media for bovine satellite cells (Will et al. 2015). Furthermore, resveratrol has been reported to induce myoblast fusion prior to differentiation (Kaminski et al. 2012), and selenium has emerged as an essential supplement for supporting the differentiation capacity of muscle cells (Bao et al. 2023).

Based on the selected growth factors and supplements identified in the literature, our aim was to develop a simple, chemically defined medium that effectively promotes the differentiation of bovine satellite cells in vitro. To achieve this, we employ a statistical design of experiments approach representing the robust and efficient optimization procedure with a data‐driven design and enabling systematic evaluation of individual components and their interactions. Potentially high number of process variables and the related complexity of involved metabolic pathways (and associated biochemistry) prompt an incremental experimental search approach for cultivation media optimization (Gilman et al. 2021). Besides, alongside the main goal of finding the optimal cultivation media, the robust and efficient optimization procedure aims to reduce the total amount of experimental runs during the optimization process. In this context, methods of statistical experimental design can be used to guide data‐driven design, characterization, and optimization (Gilman et al. 2021; Weuster‐Botz 2000). This method allows us to optimize the formulation efficiently, ensuring robust and reproducible differentiation outcomes. The final optimized serum‐free media was named MyoFusion.

## 2 Materials and Methods

### 2.1 Experimental Approach

#### 2.1.1 Isolation of Primary Bovine Satellite Cells

Primary satellite cells were isolated from the *M. supraspinatus* muscle of a 1‐month‐old Danish Holstein bull calf. Tissue samples were collected and transferred in DMEM GlutaMAX (Gibco, UK) supplemented with gentamicin (0.2 mg/mL; Biowest, France), penicillin‐streptomycin (200 U/mL and 0.2 mg/mL, respectively; Gibco, USA), and amphotericin B (5 μg/mL; Gibco, UK). Following arrival at the lab, the tissue was minced in a sterile Petri dish and enzymatically digested using a solution containing 0.25% (v/v) trypsin, 427.5 U/mL collagenase type II (Worthington Biochemical Corporation, USA), 0.01% DNase, and 1% (w/v) d‐glucose in DPBS, following the protocol described by Skrivergaard et al. (2021). Isolated primary cells were cryopreserved and designated as passage 0 (P0). All cells were pre‐plated for 4 h to reduce the amount of fibroblast before further use (Auguet‐Lara et al. 2025).

#### 2.1.2 Cell Culture and Differentiation

For seeding, P0 satellite cells were cultured in seeding medium composed of DMEM GlutaMAX (Gibco, UK), 10% heat‐inactivated fetal bovine serum (HI‐FBS; Gibco, USA), 10% horse serum (HS; Gibco, New Zealand), 1 mM sodium pyruvate, and 1% antibiotic mix (penicillin 100 U/mL, streptomycin 0.1 mg/mL, amphotericin B 2.5 μg/mL, gentamicin 0.1 mg/mL). Cells were seeded on Matrigel‐coated plates (Corning, USA) as described by Skrivergaard et al. (2021). Experiments were conducted using cells at passage 2, seeded at a density of 3000 cells per well in 96‐well plates (Thermo Scientific, Denmark).

Once cells reached ~80% confluency (after approximately 4 days), differentiation was induced by reducing the FBS concentration to 2% in DMEM GlutaMAX (control) or by switching to FBS‐free DMEM GlutaMAX for control conditions. The following factors were tested for their ability to support differentiation: fetuin (Sigma‐Aldrich, USA), bovine serum albumin (BSA; Sigma‐Aldrich, USA), insulin‐transferrin‐selenium (I‐T‐S; Gibco, USA), resveratrol (Sigma‐Aldrich, USA), dexamethasone (Sigma‐Aldrich, USA), hepatocyte growth factor (HGF; Sigma‐Aldrich, USA), cytosine arabinoside (Sigma‐Aldrich, USA), linoleic acid (Sigma‐Aldrich, USA), FGF‐II, PDGF‐BB, and TGF‐β (provided by the Department of Chemical Engineering and Applied Chemistry, University of Toronto). After 4 days of differentiation, the fusion index was analyzed to assess myotube formation.

#### 2.1.3 Cell Count and Fusion Index Analysis

Myotube staining was performed as previously described by Skrivergaard et al. (2023). Briefly, cells were fixed with 3.7% paraformaldehyde (Invitrogen, USA) for 15 min at room temperature, followed by three washes with DPBS containing calcium and magnesium (DPBS (+/+)). Cells were then permeabilized using 0.1% Triton X‐100 (Sigma‐Aldrich, USA) for 15 min and washed again three times with DPBS (+/+). Blocking was carried out using a solution of 1% (w/v) bovine serum albumin (BSA; Sigma‐Aldrich, USA) in DPBS containing 0.1% (v/v) Tween‐20 (Sigma‐Aldrich, France) for 1 h at room temperature. After blocking, cells were washed three times with DPBS (+/+) and incubated for 1 h with Alexa Fluor Plus 647 Phalloidin (Invitrogen, USA), diluted 1:300 in DPBS‐Tween 20 (0.1% v/v) containing 0.1% BSA. Hoechst 33342 (Thermo Scientific, USA) was added during the final 15 min of incubation at a 1:1000 dilution to stain nuclei.

Following staining, cells were washed three times with DPBS (+/+) and maintained in DPBS for imaging. Imaging was performed using a Cytation 5 imaging system (BioTek) with a 4× objective. Whole‐well montages were acquired and analyzed using Gen5 software. Specific parameters for myotube detections were minimum size = 60 μm, maximum size = 15,000 μm, threshold = 1500–3000, split touching objects = on and rolling ball diameter = 20 μm, and for cell count minimum size = 8 μm, maximum size = 60 μm, threshold = 5000, split touching objects = on and rolling ball diameter = 20 μm.

It should be noted that phalloidin labels filamentous actin (F‐actin), which is ubiquitously expressed in most cell types and therefore is not a myogenic‐specific marker. However, when used in conjunction with the image analysis parameters described above, such as thresholds for actin intensity, cell elongation, and multinucleation, phalloidin staining serves as a reliable surrogate for identifying myotube structures. This combinatorial approach enables the robust detection of elongated, F‐actin–rich cells with myofiber‐like morphology, making phalloidin a practical tool for high‐throughput fusion index analysis.

#### 2.1.4 Cell Culture Quantitative Traits

To evaluate the differentiation capacity of satellite cells, several morphological and quantitative traits were utilized. *Fusion index* is the most commonly used quantitative measure to determine myogenic potency (Noe et al. 2022). It is defined as the ratio of the number of nuclei within myotubes over the total number of nuclei. *Myotube area* is defined as the cytoplasmic area occupied by individual myotubes which is an indicator of myotube growth and maturation. *Cell population* is defined here as the total number of nucleis. *Myo‐nuclei* are the nuclei located within the cytoplasm of myotube, showing the degree of fusion to evaluate whether satellite cells successfully proceed through myoblast fusion and myotube formation during differentiation. *Number of myo‐nuclei per myotube* is calculated by dividing the total number of myo‐nuclei by the total number of myotubes, representing fusion efficiency. All these measures were automatically calculated in the Gen 5 software.

### 2.2 Data‐Driven Optimization Approach

In our study, we follow the statistical design of experiments approach, described in detail elsewhere (Gilman et al. 2021; Weuster‐Botz 2000; Khuri and Cornell 2018; Speed 2006; Myers et al. 2016). The basic overview of the utilized optimization procedure is as follows. At first, among the potentially high number of existent GF we pre‐select a smaller sub‐set of GFs. This should form a specific media subject to further parameters optimization. At the same stage, for each of the pre‐selected GF we determine a meaningful (in the sense of cell culture response) concentration range. The next step is to determine specific values of concentrations for each of the selected GFs allowing a best possible, evaluated in terms of some quantitative measure, bovine satellite cell differentiation in a serum‐free media.

#### 2.2.1 Selection Stage

At this stage, we experimentally evaluate a pre‐selected set of 13 growth factors (Table 1) by measuring the fusion index across media compositions defined by a specific experimental design. Each design specifies both the composition and the concentrations of the included growth factors. The number of replicates per design ranged from 4 to 12, including two control media. The exact number of repetitions was determined based on the need to achieve statistical significance, which depends on the media's response to specific growth factor concentrations.

**Table 1:** Pre‐selected growth factors with respective concentrations ranges.

Growth factor	Concentration, mg/ml	
	Min.	Max.
Fibroblast growth factor II (fbf2)	$0.2\cdot 10^{-6}$	$50.0\cdot 10^{-6}$
Platelet‐derived growth factor BB (pdgfb)	$5.0\cdot 10^{-6}$	$10.0\cdot 10^{-6}$
Hepatocyte growth factor (hgf)	$2.0\cdot 10^{-5}$	$5.0\cdot 10^{-5}$
Transforming growth factor beta (tgfb1)	$0.1\cdot 10^{-7}$	$500.0\cdot 10^{-7}$
Insulin^a^	$50.0\cdot 10^{-4}$	$870.0\cdot 10^{-4}$
Transferrin^a^	$30.0\cdot 10^{-4}$	$480.0\cdot 10^{-4}$
Selenium^a^	$30.0\cdot 10^{-7}$	$570.0\cdot 10^{-7}$
Cytosine arabinoside	$0.24\cdot 10^{-4}$	$24.0\cdot 10^{-4}$
Fetuin	$0.02\cdot 10^{-1}$	$10.0\cdot 10^{-1}$
Linoleic acid	$5.0\cdot 10^{-4}$	$50.0\cdot 10^{-4}$
Dexamethasone	$0.4\cdot 10^{-7}$	$400.0\cdot 10^{-7}$
Bovine serum albumin (BSA)	$5.0\cdot 10^{-1}$	$50.0\cdot 10^{-1}$
Resveratrol	$4.0\cdot 10^{-3}$	$6.0\cdot 10^{-3}$

^a^ Added as part of the ITS mixture from Gibco, USA.

Each design, defined by its unique combination of growth factors and their concentrations, was assessed using statistical modeling. To identify the most influential growth factors and their effective concentration ranges, we applied the following selection criteria: (i) Statistical significance: growth factors showing the highest estimated effects with statistically significant *p*‐values within the accepted model framework. (ii) biological relevance: growth factors that elicited a meaningful culture response within a defined concentration range. A response was considered meaningful if it met the criterion of statistical significance outlined in (i).

A linear approach to data modelling is considered to be sufficient for the purpose of this stage (Weuster‐Botz 2000). The linearity assumption means, no interactions between different media components $x_{i}$ occur in the range of utilized process variables, or at least they could be neglected (requires significantly smaller estimated effects for non‐linear terms compared to linear terms in a statistical model). The significant media components (variables) are determined using the regression analysis:

(1) $$y=\alpha_{0}+\sum_{i=1}^{k}\alpha_{i}x_{i}+e,$$

where y is the observations vector (fusion index); $x_{i}$ media components (growth factors); $\alpha_{i}$ regression coefficients (effects); e is the residual error, $e\sim N(0,{\sigma_{e}}^{2})$; k is the number of variables, here ${\sigma_{e}}^{2}$ is the residual error variance.

Normally, the *Plackett‐Burman* experimental design (fraction factorial design (Speed 2006; Montgomery and St 2022) is used for screening (Gilman et al. 2021). The design should provide experimentally cheap tests of multiple GF at once. This, however, requires composite (multiple‐GF) media to test. A composite media unavoidably involves possible complex interactions between growth factors which may violate the linearity assumption assumed by the data model in Equation 1 leading to unreliable (statistically insignificant) and unstable results. This is likely the case when a complex and sensitive biological system, such as bovine satellite cells, is subject to optimization. Therefore, ignoring the growth factors interactions may have severe consequences on parameters estimation especially when testing different combinations of growth factors concentrations. Technically, it may lead to statistically insignificant results of estimated effects with all consequences that follows.

We avoid the complications related to the linearity assumption by simplifying experimental design at this stage, though, considerably increase a number of experimental runs compared to the *Plackett‐Burman* design. Here we construct a single‐GF‐based media for the pre‐selected growth factors and test these media until a meaningful concentration range can be found or until its impossibility can be determined. Hence, the total number of experimental runs for different growth factors can differ significantly.

#### 2.2.2 Parameters Optimization Stage

At this stage, for the selected sub‐set of growth factors, which is expected to be much decreased compared to the initially pre‐selected number of growth factors due to the previous stage, we consider two goals. The first goal is to determine optimal concentration ranges for the selected growth factors. We achieve this by incremental moves within a search space of candidate solutions until growth factors concentrations ranges are localized around potentially optimal solution. In other words, we are going to narrow down already determined (by the previous stage) concentration ranges in order to have a sufficiently small domain that allowed good data approximation by a second‐order polynomial. The second goal is to perform such an approximation. Then we use this model to calculate optimal concentrations of growth factors that guarantee the best solution (within the specified domain). The type of optimal solution will also be characterized.

To find the optimal variable ranges, the partial factorial design (Speed 2006) is normally utilized (Weuster‐Botz 2000; Khuri and Cornell 2018; Montgomery and St 2022). The accepted assumption is that the interactions between media components and response quantities can be described by first‐order approach, which normally permits two‐components interactions (Weuster‐Botz 2000). The higher‐level interactions are part of the residual error in the model:

(2) $$y=\alpha_{0}+\sum_{i=1}^{k}\alpha_{i}x_{i}+\sum_{i=1}^{k-1}\sum_{j=2}^{k}\alpha_{\mathrm{ij}}x_{i}x_{j}+e$$

The estimated effects (regression coefficients) $\alpha_{i}$ and $\alpha_{\mathrm{ij}}$ are used to shift the variables range (concentrations of growth factors) towards higher values of the response y (fusion index); e is the residual error, $e\sim N(0,{\sigma_{e}}^{2})$; k is the number of variables. The procedure is iterated until no further improvement can be achieved. We use 4 repetitions for each experimental run.

In our problem, the search space is continuous, hence it offers an infinite number of candidate solutions. At the same time, it is impossible to perform an infinite (or even very high) number of required experimental runs due to obvious reasons. Therefore, to find an optimal solution, we need to perform data approximation by a specific model that allows straightforward selection (calculation) of an optimal solution from an infinite set of candidate solutions.

A common practice is to use the response surface method (Khuri and Cornell 2018; Myers et al. 2016). The method assumes the response variable y within the identified optimal range can be approximated by second‐order polynomial:

(3) $$y=\alpha_{0}+\sum_{i=1}^{k}\alpha_{i}x_{i}+\sum_{i=1}^{k-1}\sum_{j=2}^{k}\alpha_{\mathrm{ij}}x_{i}x_{j}+\sum_{i=1}^{k}\alpha_{\mathrm{ii}}x_{i}^{2}+e,$$

where variables defined in the same way as in the Equations 1 and 2.

The optimum then calculated using the estimated regression coefficients from the Equation 3 (Khuri and Cornell 2018):

(4) $$x^{*}=-\frac{1}{2}A^{-1}a,$$

where the matrix A and the vector a are the matrix representations of the coefficients of the model Equation 3 redefined as

(5) $$y=\alpha_{0}+\alpha^{T}x+x^{T}\mathrm{Ax}+e.$$

An optimum solution (a stationary point) can be further characterized by analyzing the eigen decomposition of the matrix A (Riley et al. 1999).

Because each media component should be examined using at least three levels (Speed 2006; Montgomery and St 2022), we use the central composite design (Montgomery and St 2022) to build the model in Equation 3. We use four repetitions for each experimental run.

## 3 Results and Discussion

### 3.1 Correlations Between Fusion Index and Other Cellular Traits of Differentiation

During various stages of the optimization process the cells responses are collected and analyzed. While any decision made at a specific optimization stage is heavily based on quantitative statistical analysis, an auxiliary support is provided by biological (qualitative) analysis of cell responses and statistically made decisions. Therefore, the purpose of the section is to give a biological insight into the system subject to optimization. That brings the necessary biological context to the entire optimization process allowing biologically guided decisions and results interpretations.

**Figure 1:**
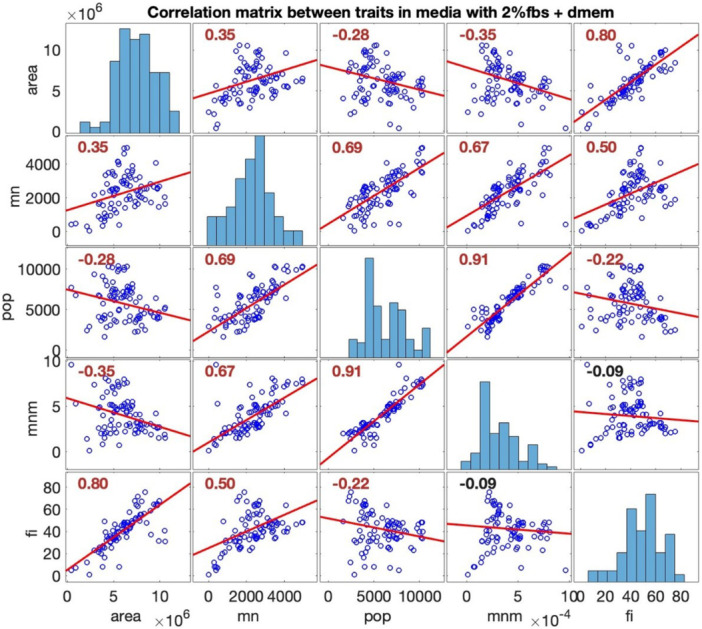
Correlations between the culture traits observed for the media with 2% FBS and DMEM. The blue circles indicate the measured data; the red lines indicate the linear fit; the numbers within each subplot indicate Pearson's correlation coefficients, where the red color highlights the significant correlations with 0.05 significance level for correlation tests. area, myotube area; fi, fusion index; mn, myo‐nuclei; mnm, number of myo‐nuclei per myotube; pop, cell population. For definition of the quantitative traits see section 2.1.4.

**Figure 2:**
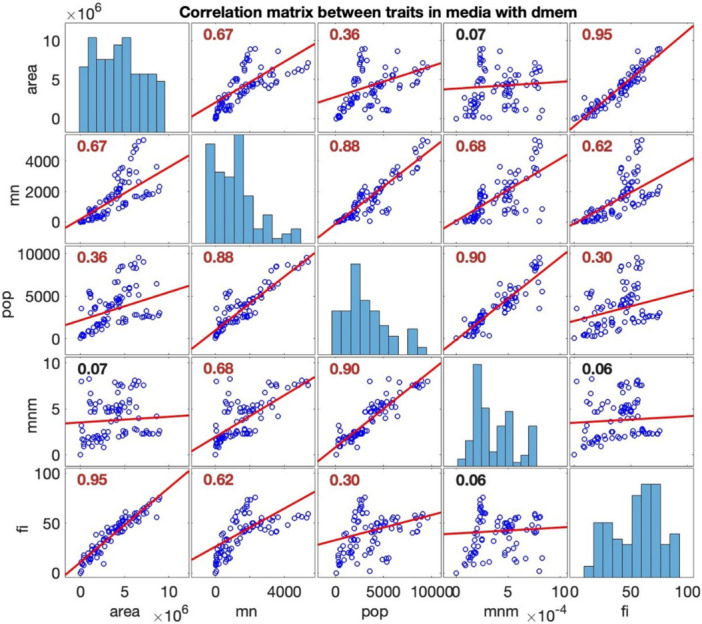
Correlations between the culture traits observed for the media with DMEM. The blue circles indicate the measured data; the red lines indicate the linear fit; the numbers within each subplot indicate Pearson's correlation coefficients, where the red color highlights the significant correlations with 0.05 significance level for correlation tests. area, myotube area; fi, fusion index; mn, myo‐nuclei; mnm, number of myo‐nuclei per myotube; pop, cell population. For definition of the quantitative traits see section 2.1.4.

To gain basic knowledge on satellite cell differentiation, we first investigated the relationship between the measured traits using the fusion index as the primary trait, both in serum and serum‐free conditions. To enable a more comprehensive interpretation of the statistical analyses associated with this optimization, we defined and monitored a set of related quantitative traits throughout the experiments. These traits characterize the culture media from multiple biological perspectives. Figures 1 and 2 provide a graphical overview of both empirical and statistical relationships among the observed traits under two control conditions.

The data indicate that myotube area and the number of nuclei within myotubes are strongly correlated with the fusion index under both serum‐containing and serum‐free conditions. Additionally, in serum‐free conditions, the total number of nuclei also correlates with the fusion index. Notably, the correlation coefficients between myotube area and nuclei count within myotubes and the fusion index are higher in serum‐free conditions than in serum‐containing conditions. This may suggest that serum‐free conditions promote differentiation compared to serum‐containing media. However, it is important to consider that the timing of analysis could influence these quantitative measurements, as demonstrated in a previous study (Skrivergaard et al. 2025).

#### 3.1.1 Differentiation Efficiency in Serum‐Free Media Is Comparable to That in Serum‐Containing Media

Serum starvation, either complete or with a low serum concentration, is widely regarded as the “gold standard” for inducing satellite cell differentiation. However, a direct comparison of these two approaches has not been thoroughly conducted for bovine satellite cells. Thus, next we evaluated the performance of serum‐free medium versus medium containing 2% FBS using ANOVA tests. Our objective was to assess the difference in satellite cell differentiation under these two conditions, based on the traits described in Section 2.1‐4. The analysis focused on comparing both the means and variances of these traits.

**Figure 3:**
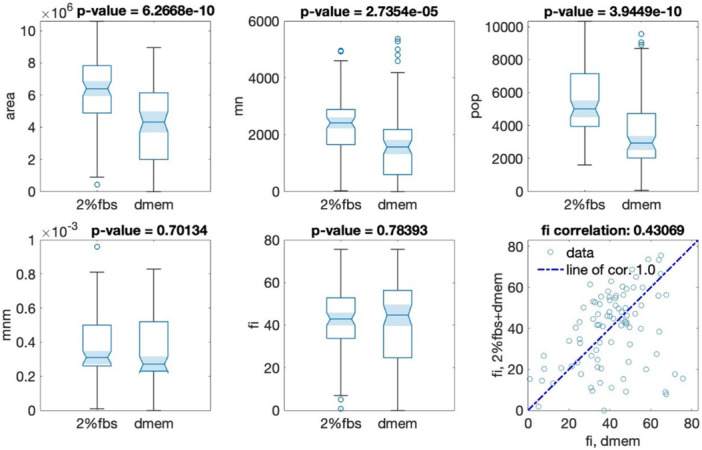
The results of ANOVA testing mean values of the media traits for the two control types. The first five (box) plots show the results of one‐way ANOVA of testing the null hypothesis that the media traits means observed at different control types are equal; the *p‐values* on the top of each plot indicate if the null hypothesis can be rejected (*p*‐value < 0.05 at the 0.05 significance level), hence, the alternative hypothesis (the two control types have different means) can be accepted; the last sub‐plot depicts correlation between the fusion index observed for the two control types. A box plot visualizes summary statistics for a specific data sample and displays in particular the median, the lower and upper quartiles, data outliers (computed using the interquartile range), and the minimum and maximum values that are not outliers. The line inside each box is the sample median. The top and bottom edges of each box are the upper and lower quartiles, respectively. The distance between the top and bottom edges is the interquartile range (IQR). The outliers represented as values that are more than 1.5*IQR away from the top or bottom of the box and are depicted as circles. area, myotube area; fi, fusion index; mn, myo‐nuclei; mnm, number of myo‐nuclei per myotube; pop, cell population. For the definition of the quantitative traits see section 2.1‐4.

The ANOVA results for trait means are shown in Figure 3. The data clearly indicate that the 2% FBS‐based medium outperforms the serum‐free medium in terms of *area*, *myo‐nuclei* count, and *population* traits, with high confidence supported by sufficiently low p‐values (top three plots in the figure). In contrast, this trend does not apply to *myo‐nuclei per myotube* and *fusion index*. Although the two media differ slightly in their means for these two later traits, the differences are not statistically significant, as reflected by relatively high p‐values (bottom two plots in the figure). Finally, the estimated correlation between the two media with respect to the fusion index is approximately 0.43.

**Figure 4:**
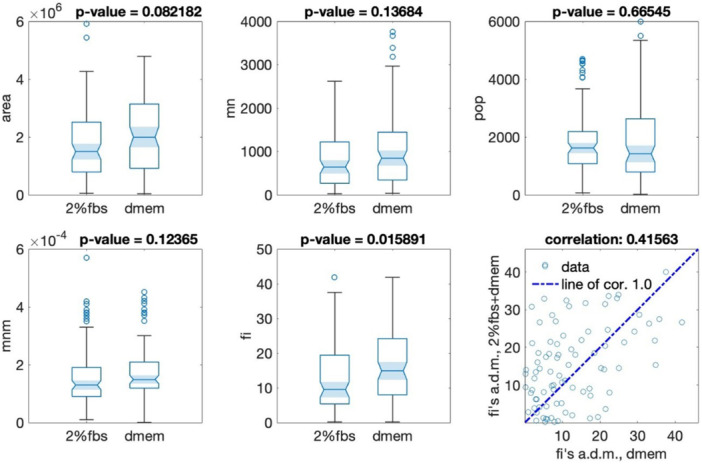
The results of ANOVA testing indicate equal variances of the media traits for the two control types. The first five (box) plots show the results of one‐way ANOVA on the absolute deviations of the media trait data values from their group means (*a.d.m*.), the Levene's test, (Derrick et al. 2018), testing the null hypothesis that the media traits *a.d.m*. observed at different control types are equal; the *p‐values* on the top of each plot indicate if the null hypothesis can be rejected (*p*‐value < 0.05 at the 0.05 significance level), hence, the alternative hypothesis (the two control types have different *a.d.m*.) can be accepted; the last sub‐plot depicts correlation between the fusion index *a.d.m*. observed for the two control types. A box plot visualizes summary statistics for a specific data sample and displays in particular the median, the lower and upper quartiles, data outliers (computed using the interquartile range), and the minimum and maximum values that are not outliers. The line inside each box is the sample median. The top and bottom edges of each box are the upper and lower quartiles, respectively. The distance between the top and bottom edges is the interquartile range (IQR). The outliers represented as values that are more than 1.5*IQR away from the top or bottom of the box and are depicted as circles. area, myotube area; fi, fusion index; mn, myo‐nuclei; mnm, number of myo‐nuclei per myotube; pop, cell population. For the definition of the quantitative traits see section 2.1‐4.

The ANOVA results for trait variances are presented in Figure 4. Prior to analysis, the data were pre‐processed using Levene's test (Derrick et al. 2018). Specifically, we calculated the absolute deviations of each trait value from its group mean and then applied ANOVA to these transformed data.

As shown in Figure 4, for most traits (except population size), the serum‐free medium exhibits greater variability compared to the 2% FBS medium. However, a statistically significant difference was observed only for the fusion index.

The results may suggest that, given the lower variation, media containing 2% FBS foster a more homogeneous cell population, in terms of developmental stage. However, that serum‐containing media do not promote higher fusion index but might increase the myotube size (area). What specific factors causing this need to be determined in future studies.

In summary, the analysis comparing differentiation in serum‐free vs. 2% FBS, demonstrated no statistically significant difference in fusion index mean between the two types of control media ($fi_{\text{serum\_free}}\;=41.91\pm 19.40$ vs. $fi_{2\%\text{fbs}}\;=42.62\pm 15.92$; *p* = 0.78). The presence of low serum levels does not appear to enhance myotube formation. Similar observations have been reported in C2C12 cells, where cultures supplemented with 2% calf serum showed no improvement compared to those maintained in F‐12 medium (Fujita et al. 2010). In that study, differentiation was assessed by analyzing the expression of developmental stage‐specific markers, such as myogenin and myosin heavy chain, rather than the phenotypic characteristics evaluated in the present work. Conversely, phase‐contrast imaging has demonstrated that C2C12 myoblasts exhibit superior differentiation when cultured in media containing 2% horse serum compared to DMEM alone (Jang et al. 2022). Notably, serum‐free conditions have also been shown to promote myotube formation more effectively than media containing 2% horse serum, as evidenced by visual assessment of myotube formation and creatine phosphokinase (CPK) activity (Lawson and Purslow 2000).

### 3.2 Development of Serum‐Free Media for Enhanced Differentiation—MyoFusion

**Figure 5:**
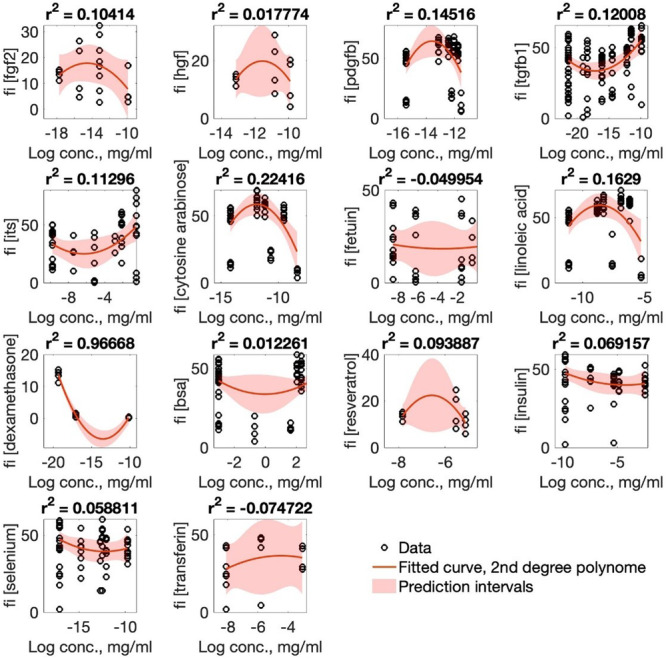
Culture response to a single‐GF‐based media expressed in terms of a fusion index. In the square brackets is specific single‐GF based media; fi indicate a fusion index; Log conc indicate the GF's concentration in the logarithmic scale; $r^{2}$ is a coefficient of determination for the second‐degree polynomial fit. fi, fusion index. For the definition of the quantitative traits, see section 2.1‐4.

The primary objective of this study was to develop a serum‐free medium that supports the differentiation of bovine satellite cells. To accomplish this, we initially conducted a preliminary screening of selected growth factors, evaluating each individually in a stepwise manner. A range of concentrations for each factor was tested based on values reported in the literature. Statistical parameters, including high mean fusion index, low *p*‐values, and high coefficients of determination, were used to identify promising candidates. Based on these criteria, three growth factors: PDGF, cytosine arabinoside, and linoleic acid, were selected for further optimization (Figure 5 and Table 2). Additionally, TGF was included in the optimization phase because, although not among the top‐ranked factors, it exhibited a relatively high mean fusion index (Table 2).

**Table 2:** Estimated effects and relevant statistics for the screened growth factors.

Growth factor	Estimate^a^			Model significance, *p*‐value	$R^{2}$
	Mean	Linear effect	Non‐linear effect		
Transforming GF beta	40.619	0.0131	—	0.06672	0.078
Platelet‐derived GF BB	50.689	4.9766	−0.5427	4.66e‐12	0.517
Cytosine arabinoside	51.078	0.7988	−0.0106	1.36e‐06	0.304
Linoleic acid	51.449	0.2496	−0.0011	2.48e‐13	0.556

^a^ The estimates are based on fusion index observations and provided using the model in Equation 2, except for the transforming GF beta where the model in Equation 1 is used.

### 3.3 Changes in the Media Traits During the Process of Parameters Optimization

Following the initial screening phase, the optimization process shifted toward narrowing the search space to enable efficient response surface modeling, ensuring accurate quadratic approximations. This was accomplished through a series of experiments designed using full factorial and central composite setups. In essence, the optimization procedure began with an initial suboptimal solution and progressively refined the results through successive experimental iterations.

**Figure 6:**
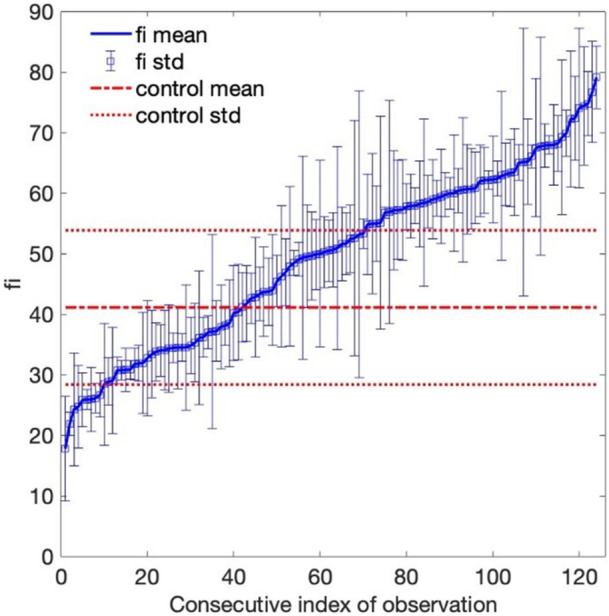
The dynamics of fusion index during the process of parameters optimization. All the data from the consecutive steps of experimental designs (starting from the full factorial, then central composite) combined together in the order it was collected (observed during experiments), hence have the assigned index of observation. Each mean value (and the related standard deviation) of fusion index is deduced from, at least, four repetitions observations. fi, fusion index. For the definition of the quantitative traits see section 2.1‐4.

The observed changes in our study are illustrated in Figure 6. The fusion index trajectory shown represents data collected across six distinct experiments, comprising a total of 120 observations (each averaged over a minimum of four replicates).

**Figure 7:**
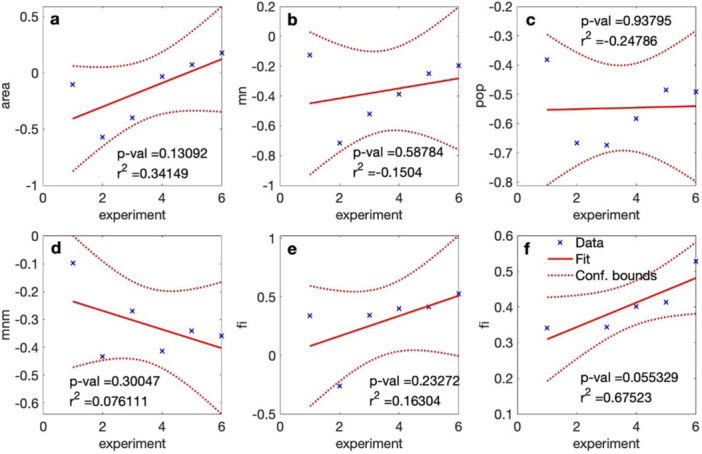
The relative changes in the media traits over the experiments. The experiments from 1 to 3 are based on full factorial designs; the experiments from 4 to 6 are based on central composite designs; crosses on the plots represent normalized traits means observed at the central points of the corresponding design; the normalized means calculated as $\bar{t}=\left(t_{i}-\bar{c}\right)/\bar{c}$, where $\bar{t}$ is normalized trait mean, $t_{i}$ trait mean at a central point, $\bar{c}$ control (2%fbs+dmem) mean; red line represent the data fit using the linear model (Equation 1) where a factor variable is an experiment (represented as its consecutive index); *p*‐val is a *p*‐value, the measure of model significance; $r^{2}$ is a coefficient of determination; the last two plots on the second row represent the same data except the result for the 2nd experiment is excluded from the very last plot (the plot no 3 counting from the left). fi, fusion index; area, myotube area; pop, cell population; mn, myo‐nuclei; mnm, number of myo‐nuclei per myotube. For definition of the quantitative traits see section 2.1‐4. (a) Area, myotube area, (b) mn, myo‐nuclei, (c) pop, cell population, (d) mnm, number of myo‐nuclei per myotube, (e) and (f) fi, fusion index.

**Figure 8:**
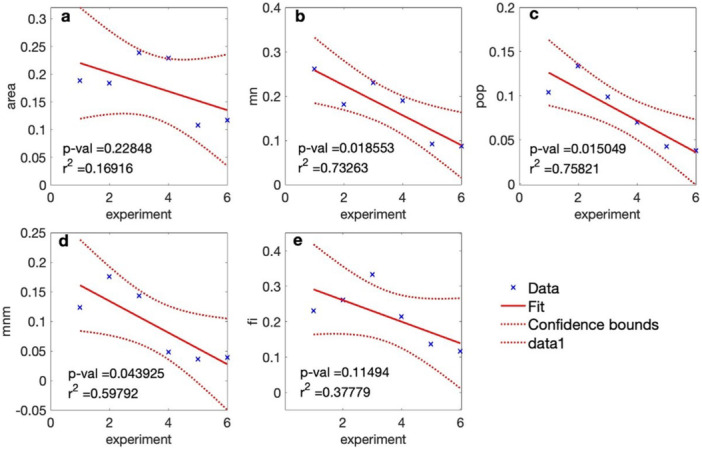
The relative changes in the media traits variability over the experiments. The experiments from 1 to 3 are based on full factorial designs; the experiments from 4 to 6 are based on central composite designs; crosses on the plots represent normalized traits standard deviations observed at the central points of the corresponding design; the normalized means calculated as $\bar{t}=\mathrm{std}(t_{i})/\bar{c}$, where $\bar{t}$ is normalized trait variability, $t_{i}$ vector of trait observations at a central point, std is the standard deviation operation, $\bar{c}$ control (2%fbs+dmem) mean; red line represent the data fit using the linear model (Equation 1) where a factor variable is an experiment (represented as its consecutive index); *p*‐val is a *p*‐value, the measure of model significance; $r^{2}$ is a coefficient of determination. area, myotube area; fi, fusion index; mn, myo‐nuclei; mnm, number of myo‐nuclei per myotube; pop, cell population. For the definition of the quantitative traits see section 2.1‐4. (a) Area, myotube area, (b) mn, myo‐nuclei, (c) pop, cell population, (d) mnm, number of myo‐nuclei per myotube, and (e) fi, fusion index.

Figure 6 provides a general overview of fusion index dynamics, presenting only basic descriptive statistics such as means and standard deviations. In contrast, Figures 7 and 8 pursue the same objective but display the outcomes of statistical modeling, incorporating the necessary inferential statistics to support reliable conclusions.

Figure 7 illustrates the sequential changes in trait means. Based on the plotted data, we can conclude with relatively high statistical confidence (*p* = 0.055, Figure 7f) that the fusion index is the only media trait that consistently increased across experiments. This trend is expected, as the fusion index served as the objective function targeted for maximization.

Figure 8 presents a similar type of plot as Figure 7 but focuses on changes in trait variability. The results indicate that the optimization procedure reduced variability in myonuclei count, cell population, and myonuclei per myotube, whereas no reduction was observed for myotube area. Regarding the fusion index, a noticeable decrease in variability was observed; however, the associated p‐value was not sufficiently low to support a statistically robust conclusion.

**Figure 9:**
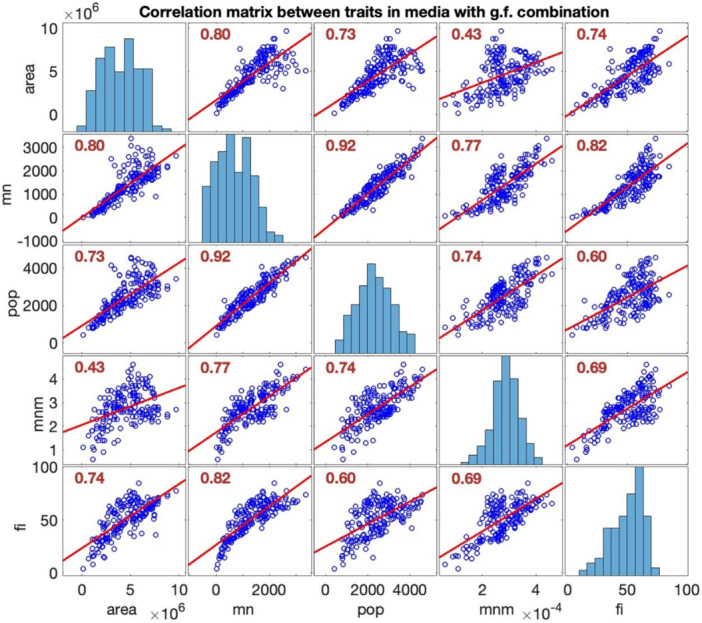
Correlations between the culture traits observed for the composite media composed with post‐selected growth factors in Table 2. The data was collected at central points of utilized experimental designs during the range of consecutive steps of parameters optimization stage. The blue circles indicate the measured data; the red lines indicate the linear fit; the numbers within each subplot indicate Pearson's correlation coefficients, where the red color highlights the significant correlations with 0.05 significance level for correlation tests. area, myotube area; fi, fusion index; mn, myo‐nuclei; mnm, number of myo‐nuclei per myotube; pop, cell population. For the definition of the quantitative traits see section 2.1‐4

Notably as showed in Figure 9, the correlation among culture traits observed in the MyoFusion medium formulated with the selected growth factors (Table 2), were high for all traits. Comparing the last rows of the Figures 1 and 2 and the last row of the Figure 9 suggesting that the MyoFusion media promotes a high degree of synchronization of the cell population. However, if this applies in large‐scale settings needs to be evaluated in detail in future studies.

The results presented in Figures 6, 7, 8, in addition to providing numerical validation of the applied experimental designs, allow several important inferences. As shown in Figures 6 and 7, the cell culture exhibits a sufficiently strong and approximately linear response to changes in medium composition. Although the variability of the fusion index decreases across experiments, these reductions are not statistically significant (Figure 8) and could potentially be reduced further.

Taken together, the linear response and remaining variability suggest that the satellite cells, even after several rounds of gradual optimization, retain the capacity to adjust their behavior in response to environmental changes. Consequently, further increases in the fusion index may still be achievable. Achieving such improvements, however, would likely require modifications to medium composition and/or a finer‐resolution exploration of the current components. This would necessitate a new experimental design, and validation of these conclusions lies beyond the scope of the present study but will be addressed in future work.

The culture's ability to respond to environmental changes is likely driven by intrinsic sources of variation. Given that all cells originated from a single donor and therefore lack genetic variation, we attribute this variability to biochemical mechanisms. Specifically, we propose that cells may utilize multiple alternative metabolic pathways whose dynamic interactions, optimized internally for performance and energy efficiency, determine the observed response phenotype. This biochemical diversity and the associated metabolic mechanisms represent a promising target for more advanced optimization studies, as they may offer substantial potential for further improving the fusion index.

### 3.4 Response Surface Analysis

For the response surface analysis (RSA), observations were collected using a central composite design and evaluated with the model described in Equation 3. In this study, we performed three separate RSAs, aiming not only to estimate an optimal parameter vector ($x^{*}$) but also to explore the search domain. This approach allowed us to empirically characterize an optimal solution within the narrowed region suggested by the full factorial design experiments.

The overall RSA results are summarized in Table 3. As shown, all three Hessian matrices contain both positive and negative eigenvalues (λ) for the same entries, indicating that all stationary points ($x^{*}$) are saddle points. Consequently, the solutions obtained are semi‐optimal rather than true optima, where $\lambda_{i}>0$ corresponds to a local minimum and $\lambda_{i}<0$ to a local maximum.

**Table 3:** The results of RSM analysis.

Growth factor	RSM 1		RSM 2		RSM 3	
	λ	$x^{*}$, mg/mL	λ	$x^{*}$, mg/mL	λ	$x^{*}$, mg/mL
Transforming GF beta	14.23	6.50e−6	10.24	12.50e−6	73.39	21.0e−6
Platelet‐derived GF BB	8.22	3.77e−6	2.97	3.40e‐6	2.59	4.66e‐6
Cytosine arabinoside	−18.99	0.66e−5	−71.79	0.73e−5	−36.37	0.73e‐5
Linoleic acid	−0.81	2.13e‐4	−1.23	2.75e‐4	−0.27	2.07e‐4

*Note:* RSM, response surface model; $\lambda ={\left[\lambda_{\mathrm{tgfb}1},\lambda_{\mathrm{pdgfb}},\lambda_{\mathrm{cyt}},\lambda_{\mathrm{lin}}\right]}^{T}$ is a vector of eigenvalues of the matrix A (Equation 5) where the elements of the vector are eigenvalues associated to a specific growth factor; $x^{*}={\left[c_{\mathrm{tgfb}1},c_{\mathrm{pdgfb}},c_{\mathrm{cyt}},c_{\mathrm{lin}}\right]}^{T}$ is a vector of concentrations, where $c_{i}$ is the concentration of the respective growth factor.

Since matrix A is symmetric, its eigenvalues are real, and its eigenvectors form an orthonormal basis (the principal axes). Each eigenvalue ($\lambda_{i}$) represents the rate of change of the fusion index along the direction of the corresponding eigenvector of A. These results suggest that optimal solutions were achieved for cytosine arabinoside and linoleic acid. However, optimality remains undefined along the directions associated with transforming growth factor beta (TGF‐β) and platelet‐derived growth factor BB (PDGF‐BB). This limitation is likely due to weak linear model performance for TGF‐β (see Figure 5 and Table 2). High variability in TGF‐β responses introduces significant uncertainty into RSA, making it difficult to identify a true optimum.

Finally, the assumptions underlying quadratic approximation are valid only if the response surface within the determined range contains a single optimum point and exhibits small gradients. The results in Table 3 suggest the presence of steep gradients and combined with the uncertainties related to TGF‐β, we cannot exclude the possibility that no optimum exists along the TGF‐β direction within the explored region.

Experimental validation of the saddle point

$x^{*}={\left[4.5\times 10^{-6},4.66\times 10^{-6},0.73\times 10^{-5},2.07\times 10^{-4}\right]}^{T}$

resulted in a semi‐optimal solution. The observed fusion index values for the composite (multi‐growth factor) medium and the local control (within the validation experiment) are:

$${\text{fi}}_{\text{GF}}=65.08\pm 4.97$$

$${\text{fi}}_{2\%\text{FBS}+\text{DMEM}}=55.63\pm 9.30$$

where ${\text{fi}}_{\text{GF}}$ and ${\text{fi}}_{2\%\text{FBS}+\text{DMEM}}$ represent the fusion index at the domain point $x^{*}$for the composite and FBS‐based media, respectively. Note that we did not use the RSA‐suggested value $x_{1}^{*}=21.0\times 10^{-6}$ due to high uncertainty caused by large variance associated with transforming growth factor beta (TGF‐β). Instead, $x_{1}^{*}=4.5\times 10^{-6}$ was chosen to remain within the experimentally tested domain, as the suggested value lies far outside the validated region.

It is worth noting that the fusion index for the control medium in this validation experiment is higher than the previously reported global mean of 42.62±15.92. The earlier value was calculated across all observations and is therefore considered a more reliable estimate. Based on these results, the optimized parameter vector $x^{*}$is expected to improve the performance of the composite medium over the FBS‐based medium by approximately 52.7% on average. A more conservative estimate, considering the upper bound of observed fusion index values for the FBS‐based medium, suggests an improvement of 11.2%.

## 4 Conclusion

In the current study we aimed at developing a serum‐free media optimized for differentiation of bovine satellite cells. This aim was concluded by developing the MyoFusion media consisting of 4.5 × 10^−6^ mg/mL TGFβ1, 4.66 × 10^−6^ mg/mL PDGF, 0.73 × 10^−5^ mg/mL Cytosine arabinoside and 2.07 × 10^−4^ mg/mL Linoleic acid in DMEM basic media.

## Author Contributions

**Aysenaz Tavsanli:** conceptualization, formal analysis, investigation, methodology, writing – original draft preparation, writing – review and editing. **Viktor Milkevych:** conceptualization, visualization, formal analysis, investigation, methodology, writing – original draft preparation, writing – review and editing. **Martin Krøyer Rasmussen:** conceptualization, funding acquisition, supervision, project administration, resources, writing – review and editing.

## Conflicts of Interest

The authors declare no conflicts of interest.

## Data Availability

The data that support the findings of this study are available from the corresponding author upon reasonable request.
